# Nanosecond phase ordering in ultra-large spin Hall nano-oscillator lattices for unconventional computing

**DOI:** 10.1038/s41565-026-02216-y

**Published:** 2026-07-10

**Authors:** Nilamani Behera, Avinash Kumar Chaurasiya, Akash Kumar, Roman Khymyn, Artem Litvinenko, Lakhan Bainsla, Ahmad A. Awad, Johan Åkerman

**Affiliations:** 1 Department of Physics, University of Gothenburg, Gothenburg, Sweden; 2 Department of Physics, School of Basic Sciences, Indian Institute of Technology Bhubaneswar, Jatani, India; 3 Research Institute of Electrical Communication (RIEC), Tohoku University, Sendai, Japan; 4 Center for Science and Innovation in Spintronics (CSIS), Tohoku University, Sendai, Japan

**Keywords:** Magnetic devices, Spintronics

## Abstract

Networks of coupled oscillators underpin fundamental studies of collective dynamics and emerging paradigms in physical computing. Spin Hall nano-oscillators (SHNOs) are particularly attractive due to their scalability and fast spin-wave-mediated interactions, yet mutual synchronization has so far been limited to small arrays and predominantly steady-state characterization. Here we demonstrate nanosecond phase ordering in lattices of up to *N* = 105,000 constriction-type SHNOs with widths of 10–20 nm. Microwave spectra reveal full mutual synchronization, a quality factor exceeding 10^6^, power scaling as *N* and linewidth scaling as *N*^−1^. Time-resolved Brillouin light scattering shows a weak, approximately logarithmic increase in the synchronization time with array size. The synchronization time varies from 10 ns in arrays of 100 SHNOs to 45 ns for the largest arrays and is consistent with Kuramoto-type collective phase-ordering dynamics in a large two-dimensional oscillator lattice. These results establish spin-wave-mediated SHNO lattices as an experimentally accessible platform for exploring collective oscillator physics and for developing embedded-Ising and reservoir-computing architectures operating at tens of gigahertz.

## Abstract (web summary)

Ultra-large networks of nano-oscillators can synchronize within tens of nanoseconds, exhibiting high coherence and offering a platform for exploring network science and developing next-generation high-speed unconventional computing.

## Main

Networks of coupled oscillators provide a universal platform for studying emergent collective dynamics^1,2^ and for implementing a variety of physical computing paradigms^3–5^. When non-linear oscillators interact through adjustable coupling, their phases can spontaneously organize into coherent states that, in suitable phase-reduced descriptions, minimize an effective energy functional, analogous to the ordering of spins in an *XY* Hamiltonian. Such synchronization processes not only illuminate fundamental aspects of non-equilibrium phase transitions, but also underpin recent efforts to realize analogue hardware for optimization, signal processing and artificial intelligence^6–9^.

Among nanoscale oscillator technologies, spin Hall nano-oscillators (SHNOs) stand out for their scalability, tunability and CMOS compatibility^10–15^. Driven by spin-orbit torques in heavy-metal/ferromagnet bilayers, SHNOs can generate microwave signals and mutually synchronize using spin waves^16–18^, which has prompted proposals for their use in Ising-type optimization^19,20^. However, previous demonstrations of mutual synchronization have been limited to arrays of up to 64 oscillators^17,21^, and little is known about the real-time dynamics of phase ordering in much larger networks, where collective behaviour could emerge on fundamentally different temporal and spatial scales. From an applications perspective, the total number of mutually synchronized oscillators *N* is critical because both microwave output power and coherence scale linearly with *N*, and sparse Ising-machine mappings typically require very large interacting networks to encode practically relevant combinatorial problems^22,23^.

In this study, we demonstrate fast, large-scale synchronization in lithographically defined SHNO lattices containing up to *N* = 105,000 constrictions with widths of 10–20 nm. To reach this regime, we combine three design strategies: (1) reducing the interoscillator distance to 24–40 nm pitch, (2) lowering the power dissipation via an energy-efficient W–Ta/CoFeB stack and (3) enhancing heat removal using thermally conductive high resistive (HiR)-Si/Al_2_O_3_ substrates^24,25^. By combining broadband microwave spectroscopy with both steady-state and time-resolved micro-Brillouin light scattering (μBLS), we directly observe nanosecond-scale phase ordering of continuous oscillator phases across micrometre-sized arrays. The synchronized emission power scales linearly with *N* while the linewidth scales inversely with *N*, yielding a record quality factor exceeding 10^6^. Time-resolved Brillouin light scattering microscopy (TR-μBLS) measurements further reveal a weak, approximately logarithmic dependence of the synchronization time on array size, from 10 ns in arrays of 100 SHNOs to reaching ∼45 ns for the largest lattices, consistent with Kuramoto-type collective phase-ordering dynamics in a large two-dimensional oscillator lattice.

## SHNO arrays

**Fig. 1:**
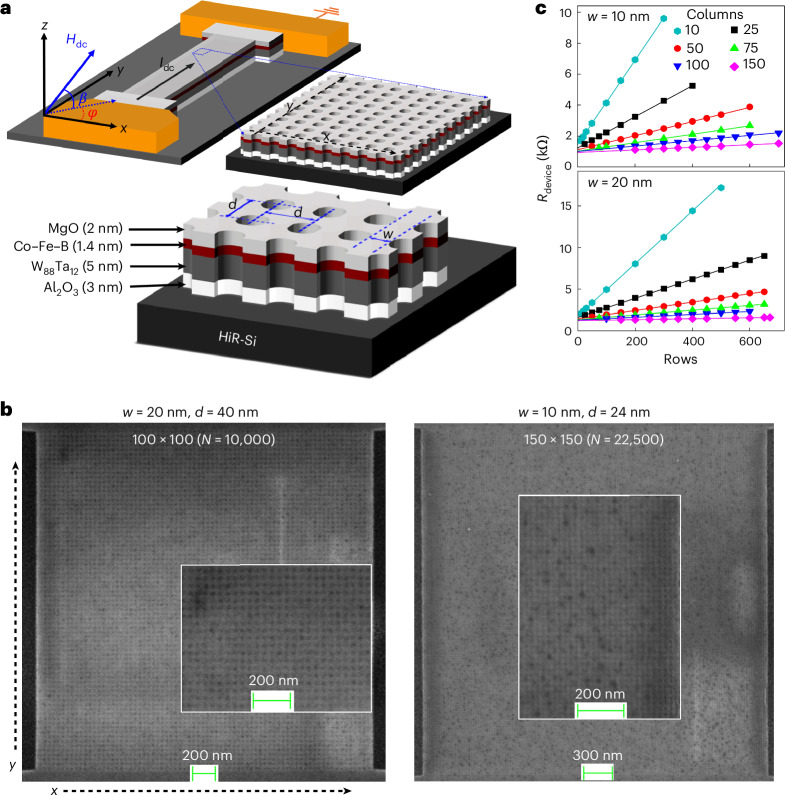
The SHNO arrays. **a**, Schematic of the SHNO arrays and their material stack, showing consecutive zoom-ins. The top cartoon shows a small part of the thick Cu/Pt contact pads (orange), the remaining part of the mesa without any nanoconstrictions (light grey) and the actual nanoconstriction array (darker grey). The directions of the drive current and the applied field are indicated. The bottom cartoon shows the material stack and the nanoconstriction width (*w*) and centre-to-centre separation (*d*). **b**, SEM images of a 100 × 100 array made from 20-nm nanoconstrictions, and a 150 × 150 array made from 10-nm nanoconstrictions. **c**, Device resistance versus number of rows for different number of columns. The solid lines are fits to equation (1) (10 nm) and equation (2) (20 nm) in the Supplementary Information. Source data

**Extended Data Fig. 1:**
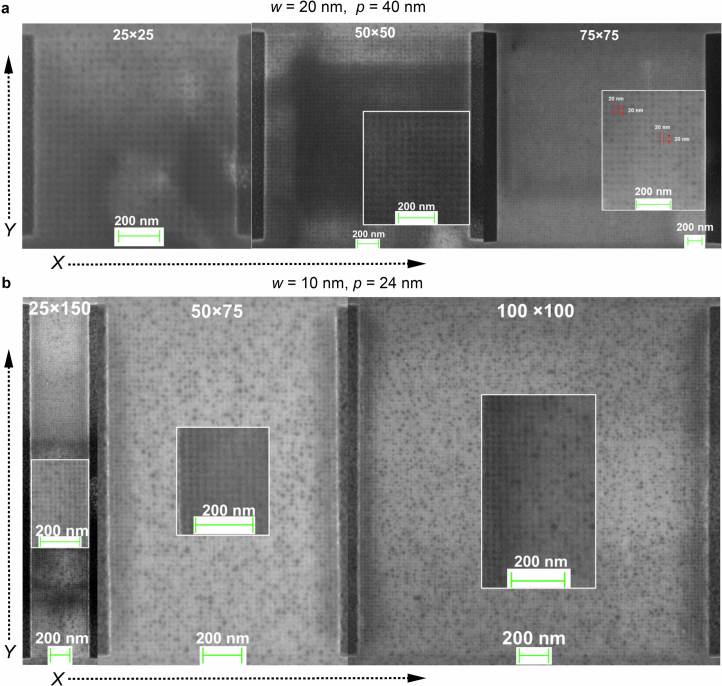
Additional SEM images of large SHNO arrays. SEM images of (**a**) 20-nm 25 × 25, 50 × 50, 75 × 75 arrays and (**b**) 10-nm 25 × 150, 50 × 75, 100 × 100 arrays. The insets highlight portions of the arrays to better discern the features of the ultra-narrow nanoconstrictions.

Figure 1a shows a schematic of a typical SHNO array device with thick Cu/Pt contact leads (orange) connecting to a patterned mesa of the material stack (grey). The lighter grey area corresponds to regions of the mesa without nanoconstrictions, while the slightly darker area indicates the actual nanoconstriction array, as seen in the first zoom-in. The final zoom-in shows the Al_2_O_3_/W–Ta/CoFeB/MgO material stack and the width (*w*) and centre-to-centre separation (*d*) of the nanoconstrictions. Figure 1b shows scanning electron microscopy (SEM) images of two square arrays: one with 100 × 100 20-nm nanoconstrictions separated by 40 nm, and the other with 150 × 150 10-nm nanoconstrictions separated by 24 nm. The inset SEM images, taken at higher resolution, highlight the good reproducibility and how the final dimensions closely match the nominal design values of *w* and *d* (see Extended Data Fig. 1 for additional SEM images). The nanofabrication and measurement details are provided in the Methods, and all measurements presented in the manuscript were performed at room temperature. As expected, the measured device resistance (Fig. 1c) scales linearly with the number of rows (*y*) and inversely with the number of columns (*x*). All resistance values are well fitted by a model accounting for the varying dimensions of the remaining mesa (see Supplementary Information, equations (1) and (2)), with the individual resistances of the 10-nm and 20-nm nanoconstrictions being 293 Ω and 348 Ω, respectively, independent of array size (Supplementary Fig. 1). The anisotropic magnetoresistance is ∼0.9% for both widths and all arrays (Supplementary Fig. 2).

## Auto-oscillations versus array size

**Fig. 2:**
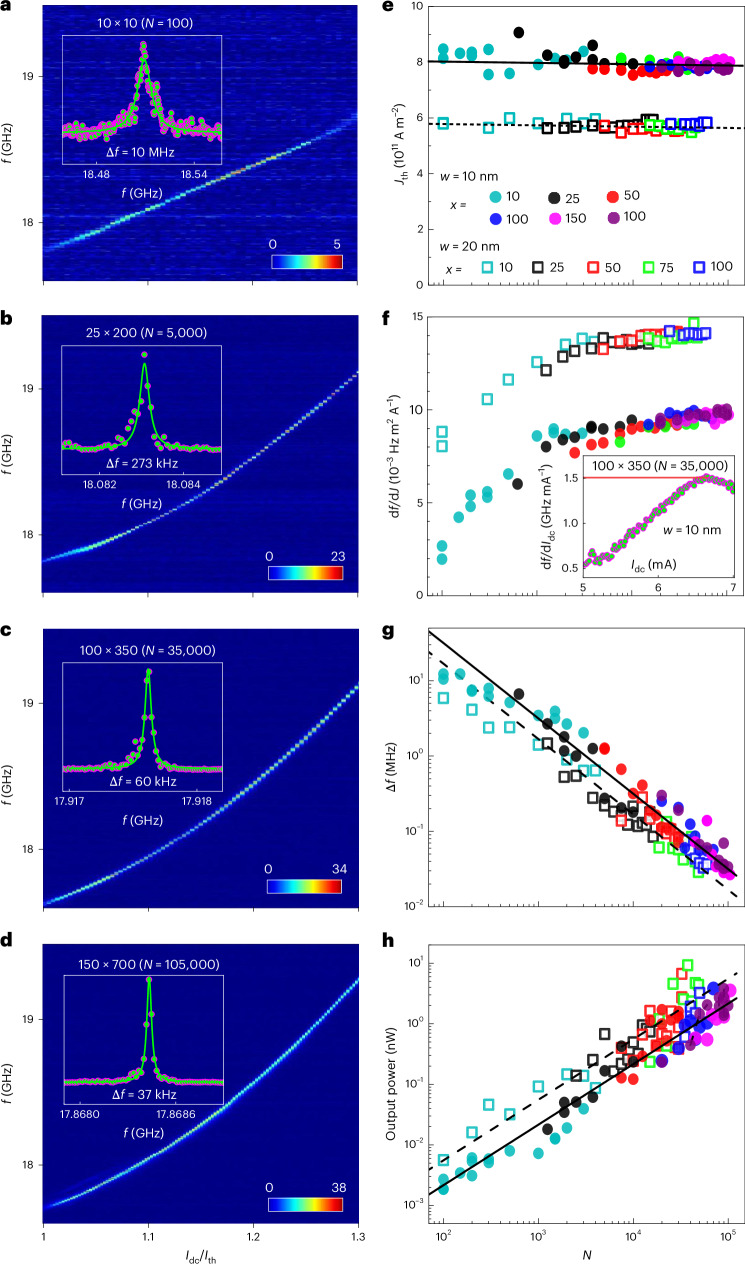
Auto-oscillation PSD, threshold current, tunability, linewidth and power. **a**–**d**, PSD versus criticality (*I*_dc_/*I*_th_) for four representative arrays with *N* = 100 (**a**), 5,000 (**b**), 35,000 (**c**) and 105,000 (**d**) nanoconstrictions (*w* = 10 nm) measured at 0.7 T, *φ* = 22° and *β* = 60°. The colour bars indicate the peak power in dB over the noise floor. Insets: individual spectra around the lowest measured linewidth (Δ*f*) for each array, together with Lorentzian fits. **e**,**f**, Threshold current density (**e**) and maximum frequency tunability (**f**) versus number of SHNOs (*N*) measured at 0.7 T (for *w* = 10 and 20 nm). Inset, **f**: maximum frequency tunability extracted from a plot of d*f*/d*I*_dc_. **g**,**h**, Linewidth (**g**) and total microwave power (**h**) versus number of SHNOs (*N*) with widths of *w* = 10 and 20 nm measured at 0.7 and 1.0 T, respectively. Solid lines (*w* = 10 nm) and dashed lines (*w* = 20 nm) are fits to *N*^−1^ in **g** and to *N* in **h**. Source data

Auto-oscillations on well-defined single microwave signals—consistent with complete mutual synchronization—were observed in all investigated arrays, with only a few arrays showing multiple signals just above auto-oscillation onset due to partial synchronization. Figure 2a–d shows the microwave power spectral density (PSD) versus normalized current (*I*_dc_/*I*_th_) of four representative 10-nm nanoconstriction arrays spanning the full array size range from 10 × 10 to 150 × 700 nanoconstrictions. Additional PSDs of 10-nm and 20-nm large SHNO arrays are shown in Supplementary Information, sections 2.1 and 2.2 (Supplementary Figs. 3 and 4). As shown in Supplementary Fig. 5, the auto-oscillation threshold current (*I*_th_) is independent of the number of rows (connected in series) and increases linearly with the number of columns (connected in parallel). As discussed in Supplementary Information, section 2.3, this scaling means that the threshold current density (*J*_th_) remains constant across all array sizes and is only slightly different between the 10-nm and 20-nm arrays (Fig. 2e), corroborating consistent reproducibility of the nanofabrication process throughout all arrays. The relative standard deviation of *J*_th_ is 2.1% in the 20-nm arrays and 3.3% in the 10-nm arrays. The auto-oscillation frequency at threshold is similarly independent of array size, and even more tightly distributed, with a relative standard deviation of 1.0% in the 20-nm arrays and 1.1% in the 10-nm arrays (Supplementary Fig. 6).

However, as can be seen in Fig. 2a–d, the positive and monotonic current dependence of the auto-oscillation frequency increases substantially with array size, showing an essentially linear and weak current dependence in the smallest arrays while becoming increasingly strong and quadratic in the larger arrays. In the inset of Fig. 2f, we plot the derivative of the frequency with respect to current for a large array, showing a linear behaviour up to a maximum, ${({\rm{d}}f/{\rm{d}}{I}_{\mathrm{dc}})}_{\max }$. We then plot (d*f*/d*J*) versus *N* for all arrays in the main part of Fig. 2f. The strong dependence of the current tunability—about a factor of five for the 10-nm arrays—scales surprisingly well with the total number of nanoconstrictions with, possibly, a very weak additional dependence on the number of columns.

For wireless communication applications, mutual synchronization offers the two equally important benefits of reducing the auto-oscillation linewidth (Δ*f*) and increasing the total microwave power. In Fig. 2g, we plot the lowest measured Δ*f* versus *N* for all the investigated arrays, with Δ*f* extracted from Lorentzian fits to the type of spectra shown in the insets of Fig. 2a–d. The solid (*w* = 10 nm) and dashed (*w* = 20 nm) lines are fits to *N*^−1^, which is the theoretically expected dependence^26–28^. As expected from theory, the linewidth of the 20-nm nanoconstriction arrays is considerably better than that of the 10-nm nanoconstriction arrays because the mode volume and energy scales approximately as *w*^2^. From the excellent scaling, we conclude that mutual synchronization is achieved in all arrays, with one of the largest 20-nm arrays (*N* = 75 × 650 = 48,750 nano-oscillators) showing the best linewidth of all arrays. Additional microwave signal analysis on this array over a wider range of magnetic field and current resulted in an exceptionally low linewidth of 25.3 kHz at an auto-oscillation frequency of 26.2 GHz, corresponding to an excellent spintronic oscillator quality factor of *Q* = *f*/Δ*f* = 1.04 × 10^6^ (Supplementary Fig. 7).

When comparing with earlier results on the ultimate linewidth of mutually synchronized SHNO arrays^17^, it is important to keep in mind that the auto-oscillation mode volume scales approximately as *V* ∝ *tw*^2^, where *t* is the ferromagnetic thickness and *w* the constriction width. The 50-nm-wide, ∼3-nm-thick Py constrictions used previously thus have a mode volume about 54 times larger than the 10-nm-wide, 1.4-nm-thick CoFeB constrictions employed here. This already explains why the maximum quality factor, although more than six times higher (*Q* ≈ 1.0 × 10^6^ versus *Q* ≈ 1.7 × 10^5^), does not scale simply with the ratio of oscillator numbers 10^5^/64. The remaining difference can be attributed to material parameters (Py versus CoFeB), the transition from predominantly localized to propagating modes in the present tightly packed arrays, and the greater sensitivity of 10-nm constrictions to edge roughness, etch damage and interface inhomogeneities.

In Fig. 2h, we plot the highest generated microwave output power versus *N* of each array together with linear fits, again showing excellent scaling. For the 10-nm arrays, the output power reaches a maximum of ∼4 nW in the 105,000-SHNO array. The highest output microwave power observed in any array is ∼9 nW for a 20-nm 75 × 500 array with 37,500 SHNOs. These observed output microwave power values are among the highest to date among all reports on SHNOs and even surpass some of the literature values achieved with magnetic-tunnel-junction-based spin-torque nano-oscillators.

## BLS microscopy

**Fig. 3:**
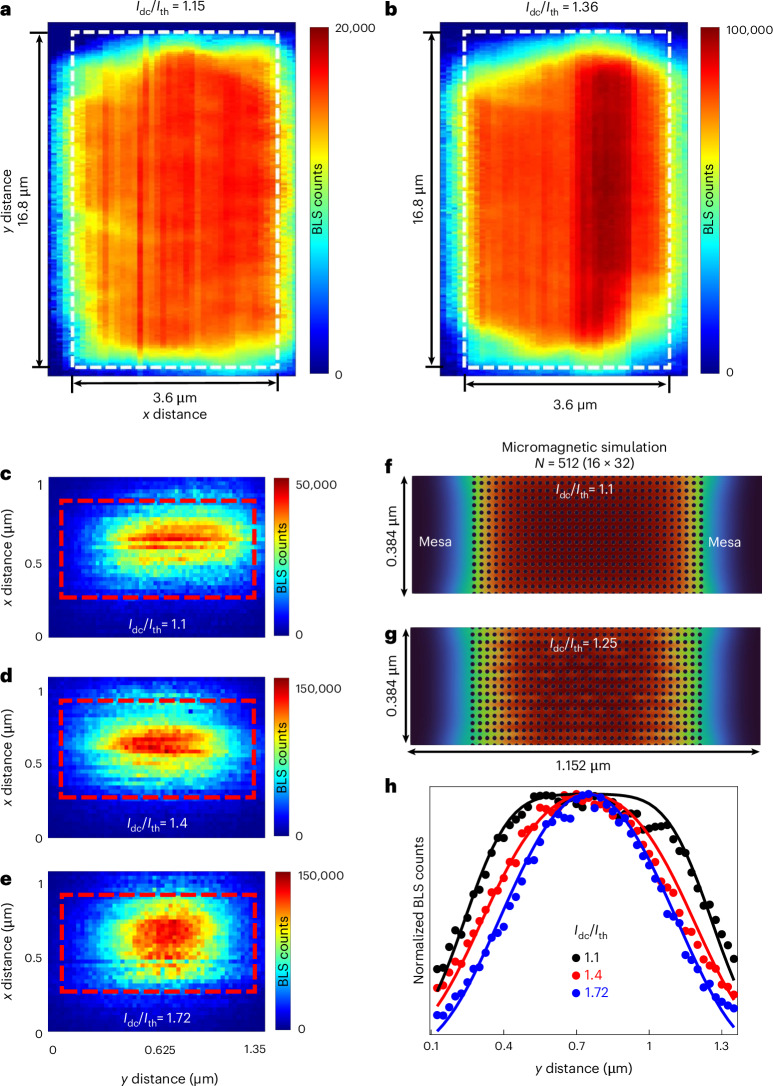
μBLS microscopy and micromagnetic simulation. **a**,**b**, μBLS microscopy images (log scale) of the largest array with 105,000 10-nm SHNOs at *I*_dc_ = 6.5 mA (**a**) and *I*_dc_ = 7.6 mA (**b**). The white dashed box indicates the extent of the array. **c**–**e**, BLS counts (linear scale) within a 25 × 50 10-nm array at increasing currents: *I*_dc_ = 1.03 mA (**c**), *I*_dc_ = 1.3 mA (**d**), *I*_dc_ = 1.6 mA (**e**). The red dashed box indicates the extent of the array. **f**,**g**, Micromagnetic simulations of a 16 × 32 10-nm SHNO array at two different currents: *I*_dc_ = 1.1 *I*_th_ (**f**) and *I*_dc_ = 1.25 *I*_th_ (**g**). **h**, Normalized BLS counts in **c**–**e** versus *y* position together with fits to a model described in the text. Source data

To directly visualize the auto-oscillations and the mutual synchronization, we use scanning μBLS microscopy and map out the spin wave intensity inside and outside of the arrays. Figure 3a,b shows BLS maps of the largest 105,000-SHNO array taken at two current levels, one just above threshold and the other 36% above threshold, where the higher current shows a five-fold increase in the maximum spin wave intensity. Close to threshold, most of the array shows a relatively uniform spin wave intensity with some fall-off towards the shorter edges. A substantial fraction of this fall-off can be ascribed to the diffraction-limited Gaussian beam profile of the BLS laser, which adds ∼300 nm of Gaussian blur and limits the spatial resolution, most easily seen along the four array edges. However, at the higher current (Fig. 3b), the fall-off becomes even more pronounced at the short edges, predominantly so towards their corners, whereas there are no corresponding changes at the long edges.

To study this unexpected behaviour in more detail, and allow for a direct comparison with micromagnetic simulations, we acquired BLS maps at increasing current for a 25 × 50 10-nm array, where the edge regions represent a larger fraction of the auto-oscillating area (Fig. 3c–e); the three current values were chosen as 10%, 40% and 72% above threshold. As can be seen, the region of highest spin wave intensity gradually shrinks towards the centre of the array, away from the short edges. We quantify this behaviour in Fig. 3h, where the data points correspond to the normalized BLS counts versus position along the *y* axis of the array and the solid lines are fits to a model discussed below. For increasing current, the relative spin wave intensity in the array clearly gravitates towards the centre.

## Micromagnetic simulations and model

We then carried out micromagnetic simulations on similarly sized 16 × 32 arrays with the same 10-nm nanoconstriction width, 24-nm separation, and using the same material and magnetic field parameters as in the experiment (Fig. 3f,g). We reproduce the propagating nature of the auto-oscillating spin wave modes, and, more importantly, also reproduce the non-uniform spin wave intensity towards the short edges, including the current-dependent focusing of the spin wave intensity towards the array centre. It is noteworthy that the simulations, unaffected by the Gaussian blur of the BLS microscope, only show a non-uniform spin wave intensity towards the short array edges. At the higher current, streaks of variable spin wave intensity on a length scale much below the spatial resolution of the BLS microscope also appear.

The non-uniform spin wave intensity can be explained by a model (Supplementary Information, section 3.2) in which each nanoconstriction emits propagating spin waves into its surroundings. Deep within the array, far away from the short edges, all nanoconstrictions exchange spin waves in equal proportions with their surrounding neighbours, resulting in a uniform spin wave intensity. However, at the short edges, spin waves are emitted into the unpatterned, non-auto-oscillating mesa, where they rapidly dissipate. The edge nanoconstrictions hence exhibit a much lower amplitude of their magnetization precession due to the loss of magnons into the mesa, and therefore also emit many fewer spin waves into their nearest nanoconstriction neighbours. Consequently, the magnon loss into the mesa is felt well into the array over a characteristic length given by the magnon decay length.

The model can also explain the unexpected gradual increase in the current tunability of the auto-oscillation frequency as *N* increases (Fig. 2f). At the auto-oscillation threshold (*J*_th_), each nanoconstriction experiences approximately the same spin wave losses, regardless of its position in the array, consistent with the independence of *J*_th_ on *N*. However, above *J*_th_, and assuming coherent mutual synchronization, the net radiative losses experienced by a nanoconstriction deep inside the array will decrease with increasing auto-oscillation amplitude due to the coherent spin waves it receives from its neighbours. To a first approximation, this reduction is linear in *J*, which transforms the linear *f* ≈ *J* dependence in single SHNOs or small arrays into an increasingly quadratic *f* ≈ *J*^2^ dependence in the larger arrays. The larger the array, the larger the effect, up to a characteristic length scale, again given by a spin wave decay length.

## TR-μBLS

For both applications and fundamental understanding, a key question is how quickly a large SHNO lattice reaches mutual synchronization once the drive current exceeds threshold. We access this collective phase-ordering time, *t*_sync_, using TR-μBLS. This stroboscopic technique measures the temporal evolution of the magnon population following a current pulse by recording the delay between the excitation pulse and the scattered photons (see Methods for details).

**Fig. 4:**
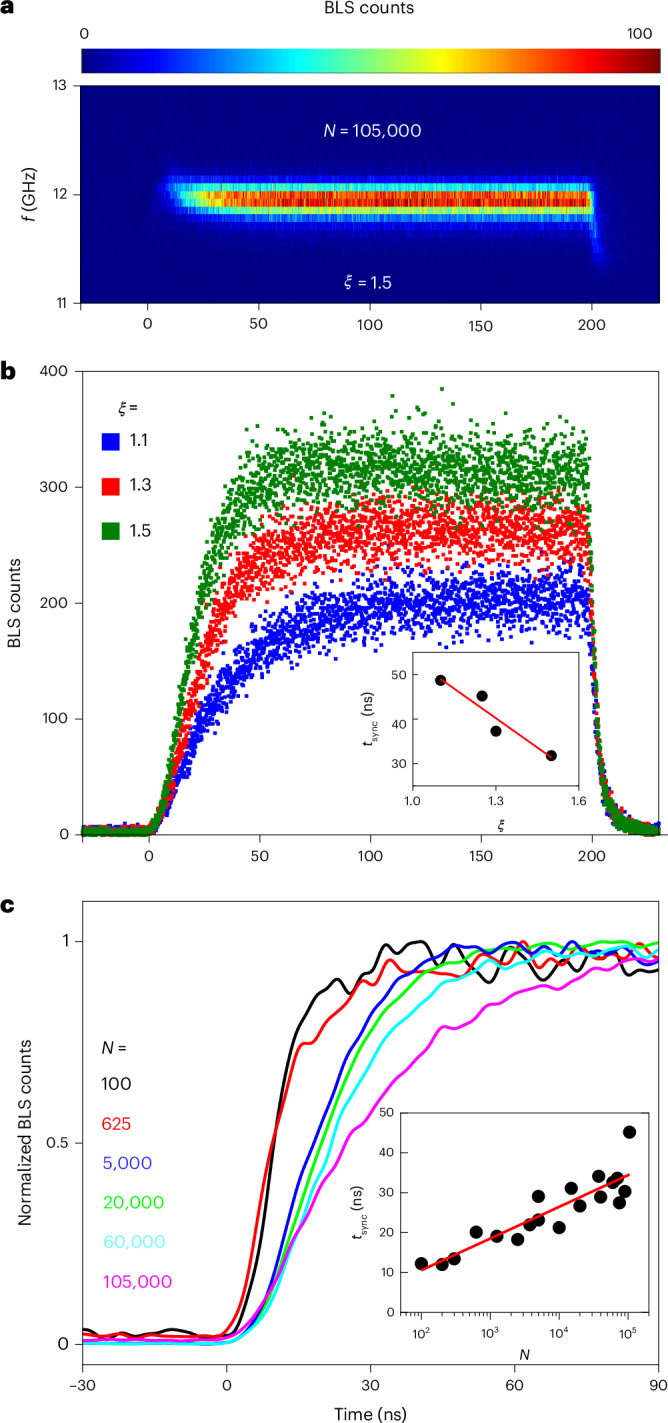
TR-μBLS of phase ordering in ultra-large SHNO lattices. **a**, TR-μBLS spectrum obtained using 200-ns current pulses for the largest array with *N* = 105,000 10-nm SHNOs, measured at a criticality *ξ* = *I*/*I*_th_ = 1.5. The colour scale shows the BLS counts on a linear scale as a function of time and frequency. **b**, Auto-oscillation frequency-integrated TR-μBLS intensity versus time for the same array at *ξ* = 1.1 (blue), 1.3 (red) and 1.5 (green). The synchronization time *t*_sync_ is defined as the 10–90% rise time of the integrated signal. Inset: *t*_sync_ as a function of *ξ* with a linear fit (red line) as a guide to the eye. **c**, Normalized and smoothed TR-μBLS intensity (*ξ* = 1.25) versus time for arrays covering the entire range *N* = 100–105,000, showing how *t*_sync_ systematically shifts to later times as *N* increases. Inset: plotting *t*_sync_ versus *N* reveals an approximately logarithmic dependence, ${t}_{{\rm{sync}}}\propto \log N$. Source data

Figure 4a shows the TR-μBLS spectrum of the largest array with *N* = 105,000 SHNOs, excited by 200-ns current pulses with criticality *ξ* = *I*_dc_/*I*_th_ = 1.5. Three regimes are observed: (1) a rapid growth of the spin-wave intensity (0–50 ns), (2) a quasi-steady saturated state (50–195 ns) and (3) a fast decay after the pulse ends. To quantify the build-up, we integrate the BLS counts over frequency and plot the resulting intensity versus time for several criticalities, *ξ* = 1.1, 1.3 and 1.5 (Fig. 4b). We define the synchronization time *t*_sync_ as the time for the intensity to increase from 10% to 90% of its saturation value, a standard measure of pulse rise time. As shown in the inset of Fig. 4b, *t*_sync_ decreases monotonically as the drive is increased. This is expected because increasing the current drives the system further above threshold, increases the auto-oscillation amplitude and strengthens the overall coupling, whether mediated predominantly by dipolar fields or by spin waves. By contrast, the decay after the pulse ends is nearly independent of *ξ* within our experimental resolution, indicating that turn-off is dominated by loss of the coherent magnon population rather than by a separate, slower desynchronization process.

We next examine how *t*_sync_ depends on the array size. Figure 4c compares normalized BLS counts for arrays with *N* ranging from 100 to 105,000 oscillators at a fixed criticality *ξ* = 1.25. The onset of synchronization systematically shifts to later times as *N* increases. Plotting *t*_sync_ versus *N* on a semilogarithmic scale (Fig. 4c, inset) reveals a linear trend, corresponding to a logarithmic dependence ${t}_{{\rm{sync}}}{\propto}{\log{N}}$, with *t*_sync_ ≈ 45 ns for the largest array.

The observed weak logarithmic size dependence is qualitatively consistent with phase ordering in two-dimensional Kuramoto-type oscillator lattices^2,3,29^. Such models have been applied to two-dimensional networks of spin-torque nano-oscillators by Flovik et al.^30^, and related studies of two-dimensional Kuramoto lattices have emphasized the role of vortices, cluster growth and finite-size synchronization^31,32^. In this picture, global synchronization proceeds through hierarchical domain growth and vortex-annihilation-like processes rather than through a purely uniform exponential build-up. Small locally synchronized domains first form, after which larger coherent regions emerge through successive merging events. If the effective synchronized domain size grows approximately multiplicatively during these merger steps, then the number of stages required to span an array containing *N* oscillators increases quasi-logarithmically with system size, giving a natural qualitative rationale for the observed ${t}_{\mathrm{sync}}\approx \log N$ dependence over the experimentally accessible range.

Although our present system does not implement programmable Ising couplings or explicit optimization tasks, the nanosecond-scale ordering across 10^5^ coupled oscillators provides a dynamic benchmark for large SHNO-based Ising networks. From the perspective of physical reservoir computing, *t*_sync_ sets a natural fading-memory timescale^33^ for the lattice: the values of 10–45 ns measured here imply that the network can respond and relax on sub-100-ns time windows, and the demonstrated tunability of *t*_sync_ with both drive and array size offers straightforward knobs to adjust the reservoir memory depth^34^ to a given task.

## Conclusions

**Extended Data Fig. 2:**
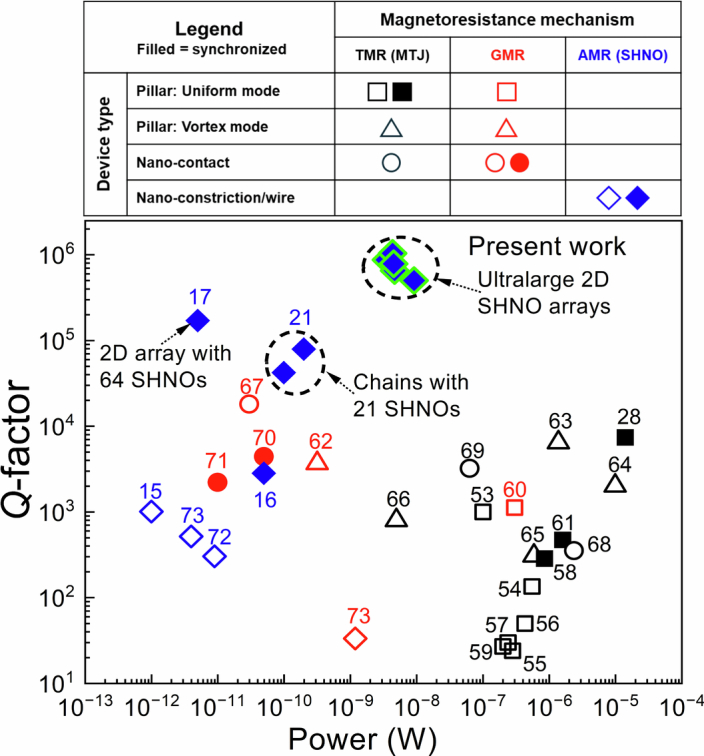
Benchmarking of spintronic oscillators. *Q*-factor versus microwave output power of various spintronic oscillators and their synchronized systems (shown with filled symbols). Different shapes and colour-coded classifications based on the type of magnetoresistance mechanisms are shown at the top. The data comprise the best performance nanopillar MTJs^28,53–61^, vortex MTJs^62–66^ nanocontact spin valves^67–71^ and SHNOs^15–17,21,72,73^. Source data

Spin-wave-based and magnonic technologies are being actively explored as candidates for energy-efficient information processing and unconventional computing beyond CMOS^35–38^. In this context, we have demonstrated mutual synchronization in SHNO lattices containing up to *N* = 105,000 nanoconstrictions, extending the size of coherent SHNO networks by more than three orders of magnitude. The synchronized arrays exhibit microwave power scaling linearly with *N* and linewidth scaling as *N*^−1^, yielding exceptional quality factors of >10^6^ and output powers up to 9 nW in the 20-nm devices (for a benchmark plot, see Extended Data Fig. 2). The combination of high output power and ultra-narrow linewidth is directly relevant for microwave applications such as wireless communication and ultrafast spectrum analysis^39–41^. TR-μBLS further shows that phase ordering occurs on nanosecond timescales, with a synchronization time that decreases with drive and increases weakly, approximately logarithmically, with array size. Together, these results establish ultra-large SHNO arrays as a model system for collective phase-ordering dynamics in large two-dimensional oscillator lattices, combining large network size, microwave operation and nanosecond phase ordering.

The square geometry of our lattices provides a natural physical substrate for the square-embedding schemes proposed for realizing fully connected Ising Hamiltonians in analogue optimization hardware^22^. Within this framework, the measured phase-ordering time sets a dynamic benchmark for how rapidly a large two-dimensional oscillator lattice could establish global coherence when used as the physical substrate of an embedded-Ising machine. Although our present experiments do not implement programmable couplings or explicit problem instances, the nanosecond synchronization across 10^5^ oscillators suggests that SHNO-based Ising architectures could, in principle, operate on extremely short timescales relative to many existing analogue platforms.

At the same time, the rich non-linear transient response, intrinsic short-term memory and high dimensionality of the SHNO lattice make it a promising medium for physical reservoir computing^6–8^. The nanosecond-scale dynamics and gigahertz operating frequencies imply an exceptionally large processing bandwidth, while the tunable non-linearity and coupling strength provide natural knobs for task adaptation. Future work could harness these properties by encoding time-dependent inputs in the drive current or magnetic field, reading out spatially resolved microwave or optical signals, and applying task-adaptive training protocols analogous to those recently demonstrated in other physical reservoirs^42^. In this perspective, the present results define the dynamic operating regime—speed, coherence and scalability—on which such SHNO-based reservoirs could be built.

More broadly, the fact that all fabricated arrays exhibited robust mutual synchronization suggests that the upper size limit for coherent SHNO networks has not yet been reached. Further reductions in constriction spacing, increases in magnetic thickness, engineering of multilayer stacks or alternative array geometries beyond the square lattice^43^ could enable even larger, more strongly coupled and functionally richer networks. In addition, SHNO coupling and frequency can be tuned electrically via gate voltages and memristive control^44–47^, providing a natural route toward programmable interaction strengths and local biases that are required in practical embedded-Ising and reservoir-computing architectures. Ultra-large SHNO arrays thus offer a scalable and energy-efficient platform not only for prospective computing applications, but also for fundamental studies of non-equilibrium phase transitions, defect dynamics and network phenomena in driven oscillator lattices at the nanoscale^48^.

## Methods

### Material growth

Al_2_O_3_(3 nm)/W_88_Ta_12_(5 nm)/Co_20_Fe_60_B_20_(1.4 nm)/MgO(2 nm) material stacks were magnetron sputtered onto high-resistance intrinsic (undoped) silicon substrates, working at a base pressure of 2.5 × 10^−8^ torr, while the argon pressure during sputtering was maintained at 3 mtorr for all layers. A 4-nm Al_2_O_3_ capping layer was added to protect the MgO from moisture. The growth rates of W_88_Ta_12_, Co_20_Fe_60_B_20_, MgO and Al_2_O_3_ layers were maintained at 0.11, 0.15, 0.04 and 0.03 Å s^−1^, respectively, for good thickness control. The stacks were subsequently annealed at 300 °C for 60 min at the chamber’s base pressure to crystallize both MgO and CoFeB at the interface. All key sputtering parameters (base pressure, argon pressure and growth rates) followed those used in our previous works, where the W–Ta alloy and the Al_2_O_3_ seed layer were optimized for minimal heat generation, maximum heat conduction and lower current shunting^24,25^.

### Design and fabrication of square and rectangular SHNO arrays

Nanoconstriction SHNOs of two widths, *w* = 10 and 20 nm, were fabricated using electron-beam lithography (EBL). A total of 146 different square and rectangular SHNO arrays with different numbers of columns (*x* = 10–150) and rows (*y* = 10–1,000) were defined in the centre of 6 × 22 μm^2^ mesas for the 10-nm SHNOs and 8 × 30 μm^2^ mesas for the 20-nm SHNOs. The centre-to-centre separation of the 10-nm SHNOs was *d* = 24 nm, while that of the 20-nm SHNOs was *d* = 40 nm. Between the SHNO arrays, we also fabricated microbars of three different sizes (6 × 12, 6 × 18, 6 × 22 μm^2^) for spin-orbit torque characterization using spin-torque ferromagnetic resonance (ST-FMR) measurements.

EBL proceeded by covering the material stack with negative resist (HSQ, XR-1541-002, 2%) followed by EBL exposure (Raith EBPG 5200). Argon-ion beam etching was done using an Oxford Ionfab 300 Plus etcher. An optical lithography lift-off process defined ground–signal–ground (GSG) co-planar waveguides in a sputter-deposited Cu (800 nm)/Pt (20 nm) bilayer. A Zeiss Supra 60 VP-EDX scanning electron microscope was used to inspect the quality and dimensions of all SHNOs and arrays.

Extended Data Fig. 1 shows SEM images of 20-nm SHNO arrays with *x* × *y* being 25 × 25, 50 × 50, 75 × 75 (Extended Data Fig. 1a) and 10-nm SHNO arrays with *x* × *y* being 25 × 150, 50 × 75, 100 × 100 (Extended Data Fig. 1b). The insets show parts of the same devices at higher magnification.

### Electrical characterization

The resistance and anisotropic magnetoresistance of all SHNO arrays were extracted from two-point resistance measurements of microstrip devices in a vector magnet with a rotatable in-plane field of 0.25 T. An in-plane field angle *ϕ* = 0° was defined as the current and magnetic field directions being perpendicular (minimum resistance).

Magnetization auto-oscillation measurements were carried out using a custom-built probe station. The device under test is mounted inside an electromagnet on a holder that allows for variable in-plane and out-of-plane field angles. GSG probes connect to the corresponding GSG pads of the device and d.c. current is fed to the device through the d.c. port of a high-frequency bias-T. A 31-dB low-noise microwave amplifier (with a noise figure of 2.5 dB) connected to the high-frequency output of the bias-T amplifies the auto-oscillation microwave voltage before it is recorded by a Rohde *&* Schwarz FSV40 spectrum analyser. The reported microwave signal strength is given after correcting for the impedance mismatch between the SHNO array and the 50-Ω measurement system.

### μBLS measurement

A monochromatic continuous-wave laser (wavelength, *λ* = 532 nm; laser power, *P*_power_ = 0.2 mW) was focused onto the samples by a ×100 microscope objective with a large numerical aperture (0.75) down to a 300-nm diffraction-limited spot diameter. The external magnetic field was applied at an in-plane angle (*φ*) of 22° and an out-of-plane angle (*β*) of 60°, and a constant magnetic field magnitude of 0.5 T. The inelastically scattered light from the sample was collected by the same microscope objective and analysed with a Sandercock-type six-pass tandem Fabry–Pérot interferometer (TFP-1, JRS Scientific Instruments). Stabilization software based on an active feedback algorithm (THATec Innovation) was used to achieve long-term spatial stability during the μBLS measurement. The experimentally obtained BLS intensity is proportional to the square of the amplitude of the dynamic magnetization.

To investigate the characteristic synchronization time, TR-μBLS measurements were performed on a few selected large-array SHNOs. TR-μBLS is a stroboscopic technique based on the principle of time-of-flight, and captures the temporal delay between the excitation pulse and the scattered photons from magnons^49–52^. To construct the TR-μBLS spectra directly in the frequency domain, four signals are required, all of which are recorded by Time Tagger channels (Swabian Instruments). The first channel records the start of the measurement triggered by a digital delay generator (SR DG645, Stanford Research Systems). The second channel receives the photon-counting signal from the TFP-1 interferometer. Channels 3 and 4 are connected to the BLS DAQ hardware which provides the frequency information. All space- and time-resolved μBLS measurements were performed at room temperature.

### Benchmarking of spintronic oscillators

Extended Data Figure 2 shows the benchmarking of *Q*-factor versus output power for various types of spintronic oscillators based on their magnetoresistance mechanism. This includes the best performance nanopillar magnetic tunnel junctions^28,53–61^, vortex magnetic tunnel junctions^62–66^, nanocontact spin valves^67–71^ and SHNOs^15–17,21,72,73^.

## Online content

Any methods, additional references, Nature Portfolio reporting summaries, source data, extended data, supplementary information, acknowledgements, peer review information; details of author contributions and competing interests; and statements of data and code availability are available at 10.1038/s41565-026-02216-y.

## Supplementary information

Supplementary Information Supplementary Notes 1–3, Discussion, Figs 1–8 and References.

## Source data

Source Data Fig. 1 Source Data of device resistance versus number of rows for different number of columns.

Source Data Fig. 2 Source Data of auto-oscillation power spectral density, threshold current, tunability, linewidth, and power.

Source Data Fig. 3 Source Data of normalized BLS counts versus *y*-position.

Source Data Fig. 4 Source Data of time-resolved micro-Brillouin light scattering.

Source Data Extended Data Fig. 2 Source Data of Benchmarking plot.

## Data Availability

The data supporting the results in this study are available in the paper and in its Supplementary Information. Source data are provided with this paper and are available via Zenodo at 10.5281/zenodo.20053131 (ref. ^74^). All other data supporting the conclusions of this work are available from the corresponding authors upon reasonable request. Source data are provided with this paper.

## Code availability

The codes used to perform the micromagnetic simulation in this work are available via Zenodo at 10.5281/zenodo.20053131 (ref. ^74^).
