# Supplementary material for: Nanosecond phase ordering in ultra-large spin Hall nano-oscillator lattices for unconventional computing
Source: Nat Nanotechnol. 2026 Jul 10;21(8):1105–12. doi: 10.1038/s41565-026-02216-y (PMC13468112; doi:10.1038/s41565-026-02216-y)
Supplement: Supplementary file 1 [file 41565_2026_2216_MOESM1_ESM.pdf]

nature nanotechnology

Supplementary information https://doi.org/10.1038/s41565-026-02216-y

Nanosecond phase ordering in ultra-large spin Hall nano-oscillator lattices for unconventional computing

In the format provided by the authors and unedited

Contents

1 Transport properties of the SHNO arrays **S2**

1.1 Resistance . . . S2

1.2 Anisotropic magneto-resistance . . . S3

2 Auto-oscillation measurements **S4**

2.1 Additional electrical microwave PSDs of 10-nm SHNO arrays . . . S4

2.2 Representative electrical microwave PSDs of 20-nm SHNO arrays . . . S4

2.3 Threshold current and threshold current density . . . S4

2.4 Frequency tunability . . . S7

2.5 Record-high Quality factor . . . S9

2.6 Record-high SHNO auto-oscillation frequency . . . S10

3 Simulations and modeling **S11**

3.1 Micromagnetic simulations . . . S11

3.2 The model of the magnon transfer . . . S11

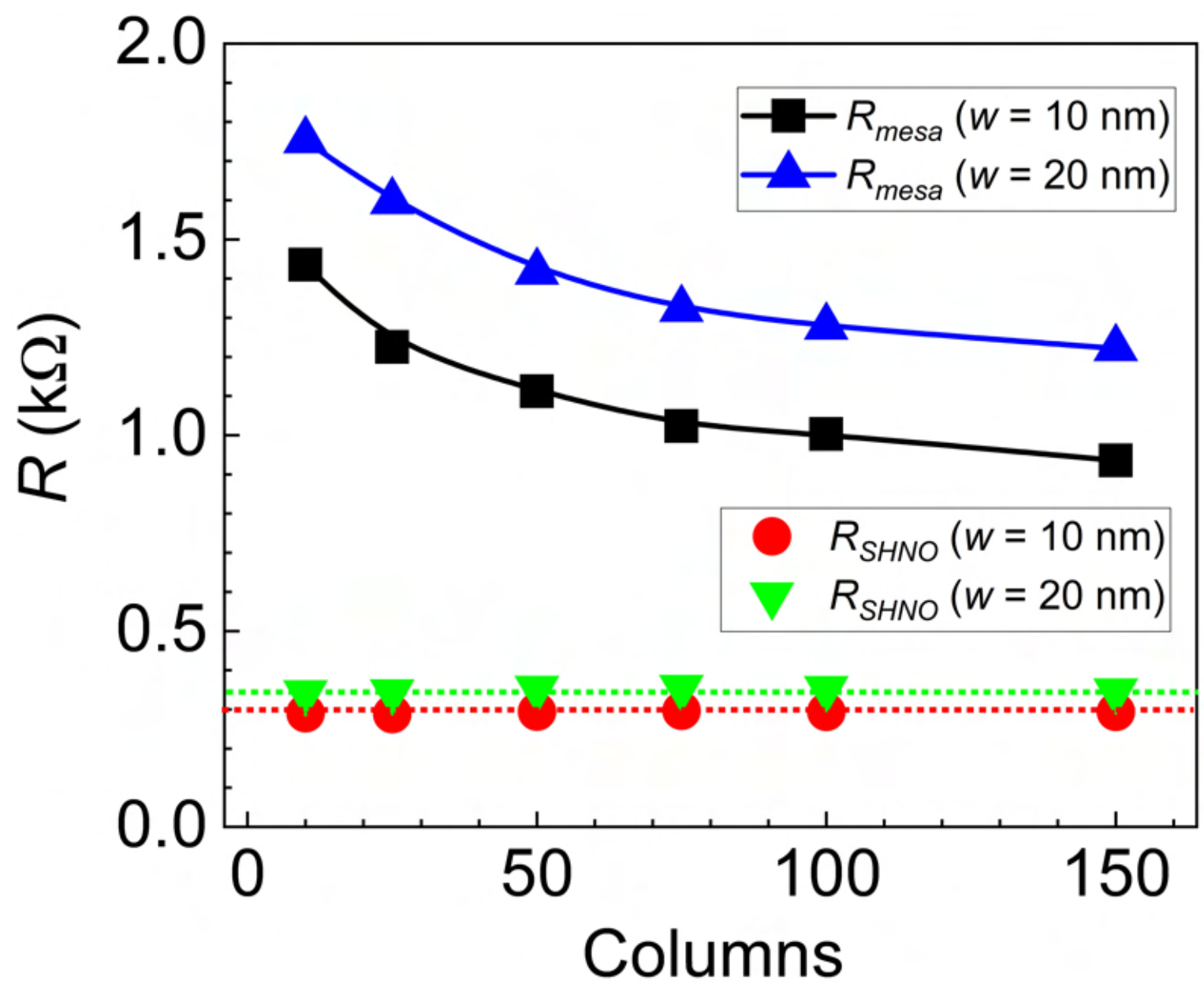

Figure S1: **Device resistance parameters.** From the measurement of device resistance, R_{mesa} & R_{SHNO} $vs.$ number of Columns for *w* = 10-nm and 20-nm constriction width SHNOs estimated by using Eqs. S1 & S2, respectively (R_{device} $vs.$ Rows shown in Fig. 1 of the main text).

1 Transport properties of the SHNO arrays

1.1 Resistance

The total resistance of a device with an SHNO array (R_{device}) can be approximated by the series resistance of the SHNO array itself and the resistance of the mesa between the array and the thick contact pad metal. Without SHNOs, the length of the mesa between the thick contacts is 20 μm and 28 μm for the 10 and 20-nm SHNOs respectively; we define R_{mesa} as the resistance of this mesa without SHNOs. As every row of 10-nm SHNOs subtracts 0.024 μm from the mesa, and every row of 20-nm SHNOs subtracts 0.040 μm, the resistance of the remaining mesa will be $(1 - 0.0012 * \mathrm{Y})R_{mesa}$ and $(1 - 0.00143 * \mathrm{Y})R_{mesa}$, respectively. The total device resistance is then given by

$$R_{device} = (1 - \mathrm{Y} * 0.0012)R_{mesa} + \frac{\mathrm{Y}}{\mathrm{X}} \times R_{SHNO}, \text{ for } w = 10 \text{ nm}, \tag{S1}$$

$$R_{device} = (1 - \mathrm{Y} * 0.00143)R_{mesa} + \frac{\mathrm{Y}}{\mathrm{X}} \times R_{SHNO}, \text{ for } w = 20 \text{ nm}, \tag{S2}$$

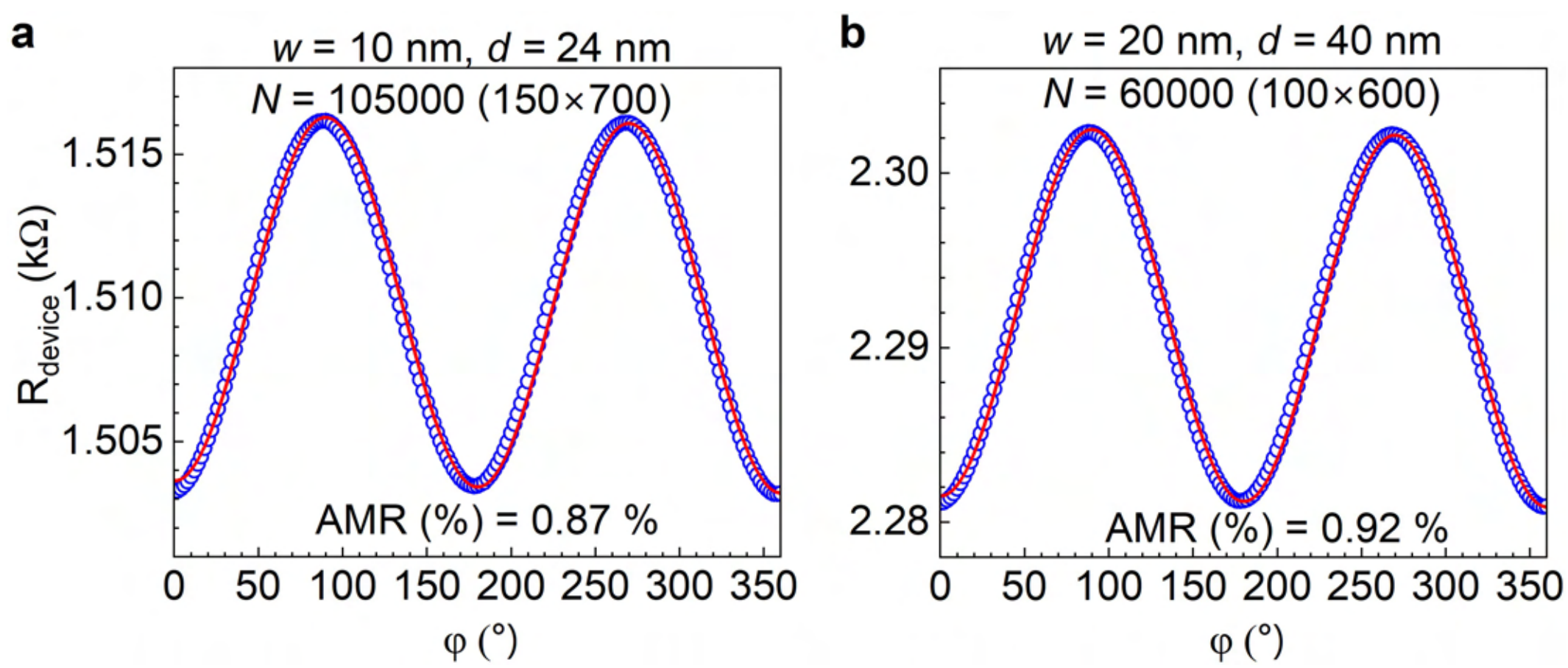

Figure S2: **AMR measurements. a** & **b**, in-plane angle dependent resistance measurements at a fixed applied field of 0.25 T and a current of I_{dc} = 0.5 mA for two large SHNO arrays with parameters given in the plots. The red solid lines are sinusoidal fits and the extracted AMR is shown underneath. All measured arrays showed an AMR of approximately 0.9 %.

where R_{SHNO} is the resistance of each nano-constriction, and X and Y are the number of columns and rows, respectively. As shown in Fig. 1b of the main manuscript, the resistance of all arrays, from the smallest 10 $\times$ 10 to the largest 105 $\times$ 700, is well fit by Eqs. S1 & S2, indicating consistent with the reproducibility of the EBL patterning. Fig. S1 shows the estimated plot of R_{mesa} & R_{SHNOs} vs. Columns, for the w = 10-nm and 20-nm arrays. The resistance of each nano-constriction SHNO stays the same regardless of the array layout: 293 Ω and 348 Ω for the 10-nm and 20-nm nano-constrictions, respectively. Interestingly, the mesa resistance decreases with the number of columns in the array, reflecting how the current makes better use of the full mesa when the width of the SHNO array approaches that of the mesa.

1.2 Anisotropic magneto-resistance

Figure S2 (a & b) show measurements of the in-plane angle-dependent resistance for two of the largest arrays measured in an applied field 0.25 T and I_{dc} = 0.5 mA. The corresponding anisotropic magneto-resistance (AMR)(%) values are estimated after fitting these curves (red solid lines) by using the equation:

$$R_{device}(\varphi) = R_{\perp} - [\Delta R \times \cos^2(\varphi)] \tag{S3}$$

where, $R_{device}(\varphi)$ is the measured device resistance, $R_{\perp}$ is resistance when both I_{dc} and applied are in-plane perpendicular direction, $\Delta R = R_{\perp} - R_{\|}$. From the extracted fitting parameters, the AMR(%) is estimated by using the relation: $(\Delta R/R_{\perp})\times$ 100 and its value approximately 0.9 % for both SHNO widths.

2 Auto-oscillation measurements

2.1 Additional electrical microwave PSDs of 10-nm SHNO arrays

Figure S3 (a-f) show the additional PSDs for 10-nm SHNO arrays with varying $X\times Y$ (where X = 10-150 & Y = 100-1000) measured at fixed H_{dc} = 0.7 T, φ = 22°, and β = 60°. From these, many important conclusive plots have been derived and shown in Fig.2 of the main text.

2.2 Representative electrical microwave PSDs of 20-nm SHNO arrays

In addition to the PSDs of 10-nm SHNO arrays, we also performed similar PSD measurements for all 20-nm SHNO arrays at different applied field in the range of 0.5 to 1.1 T. Figure S4 (a-e) show the representative PSDs of 20-nm SHNO arrays measured at similar orientations consistent with 10-nm arrays with at different applied magnetic field in the range of 0.7-1.0 T. From the PSDs similar extracted information has been discussed in Fig.2 of the main text.

2.3 Threshold current and threshold current density

As shown in Fig. S5a, the auto-oscillation threshold current (I_{th}) is extracted from plots of the inverse of the integrated power *vs.* current [1] of each microwave PSD signal of the type shown in Fig. S3 & S4. The linear fit is applied to the first few data points above threshold, *i.e.* the current range where the inverse integrated power remains approximately linear; the exact number of points ($\sim$ 3–5) varies slightly from array to array depending on signal quality.

In Fig. S5b, the extracted I_{th} *vs.* N is then plotted for all arrays, showing how I_{th} is independent of the number of rows (Y) but scales linearly with the number of columns (X).

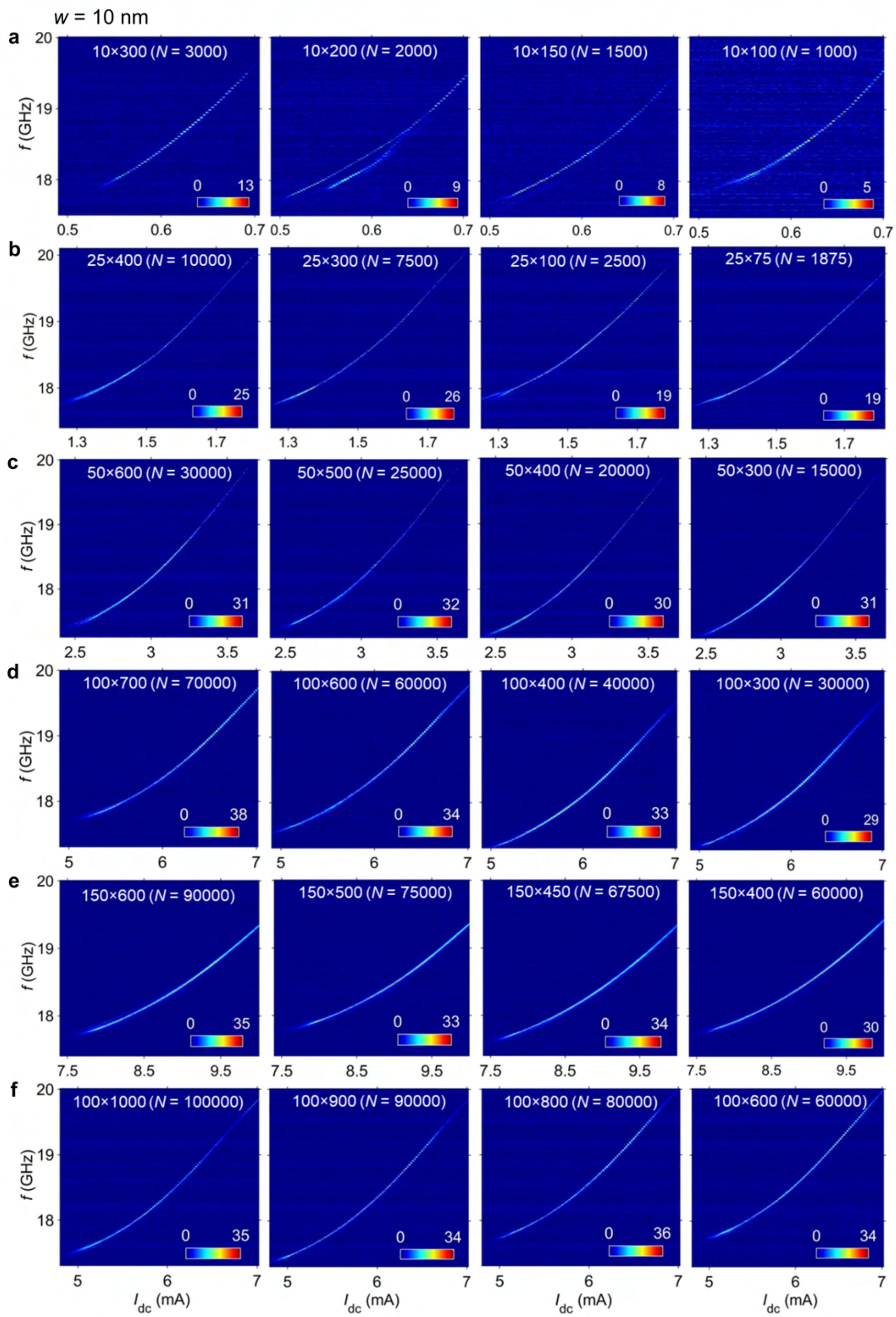

Figure S3: **Additional electrical PSD plots of 10-nm SHNO arrays.** **(a)** 10 × (300-100), **(b)** 25 × (400-75), **(c)** 50 × (600-300), **(d)** 100 × (700-300), **(e)** 150 × (600-300), and **(f)** 100 × (1000-600) no. of SHNOs measured at H_{dc} = 0.7 T, φ = 22°, and β = 60°. The inset scale bar in each PSD indicates the dB/Noise.

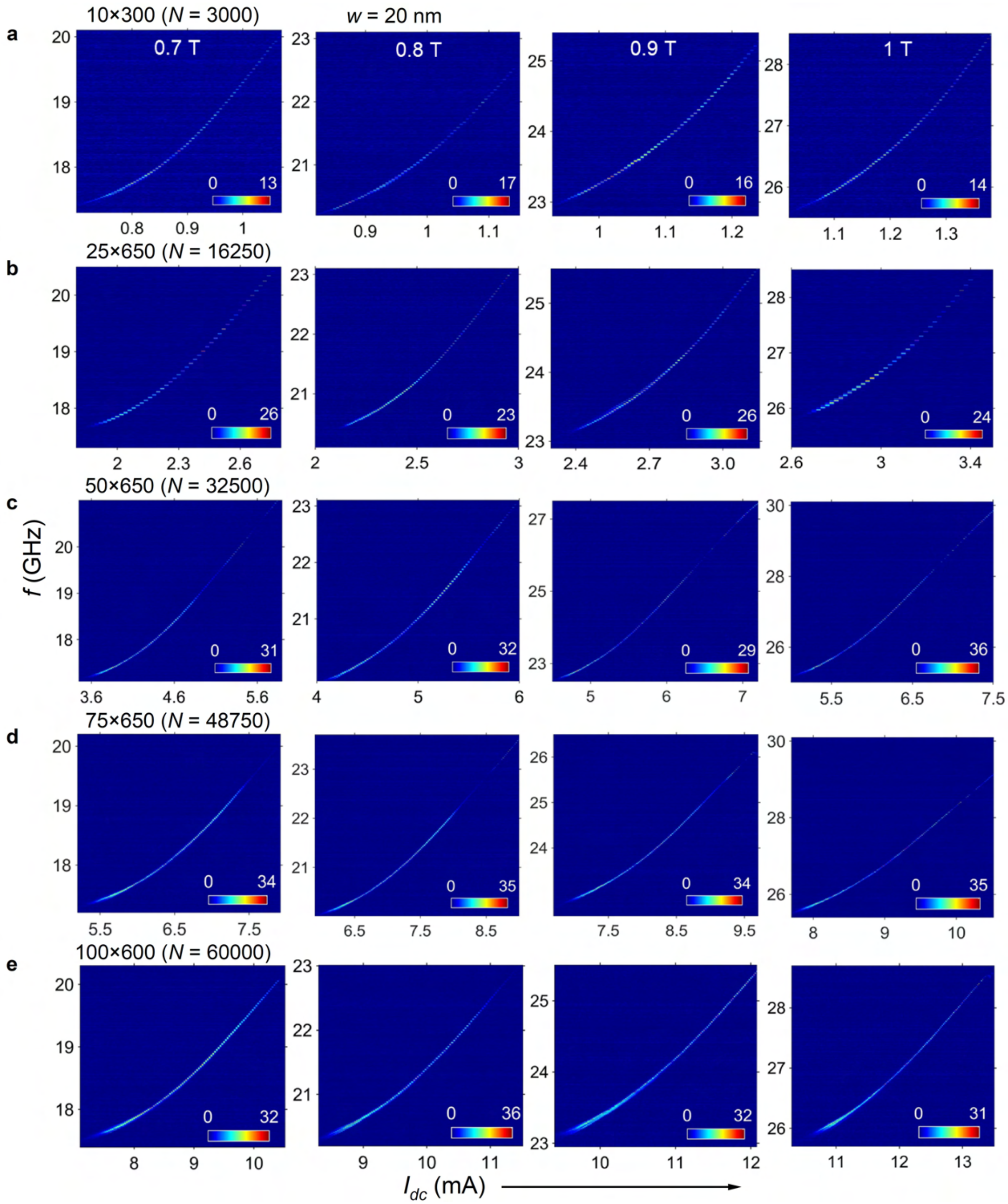

Figure S4: **Field dependent representative electrical PSDs of 20-nm SHNO arrays. (a-e)** The field-dependent PSDs of $X \times Y$ based large array SHNOs measured at 0.7-1 T, φ = 22°, β = 60°. The inset scale bar in each PSD indicates the dB/Noise.

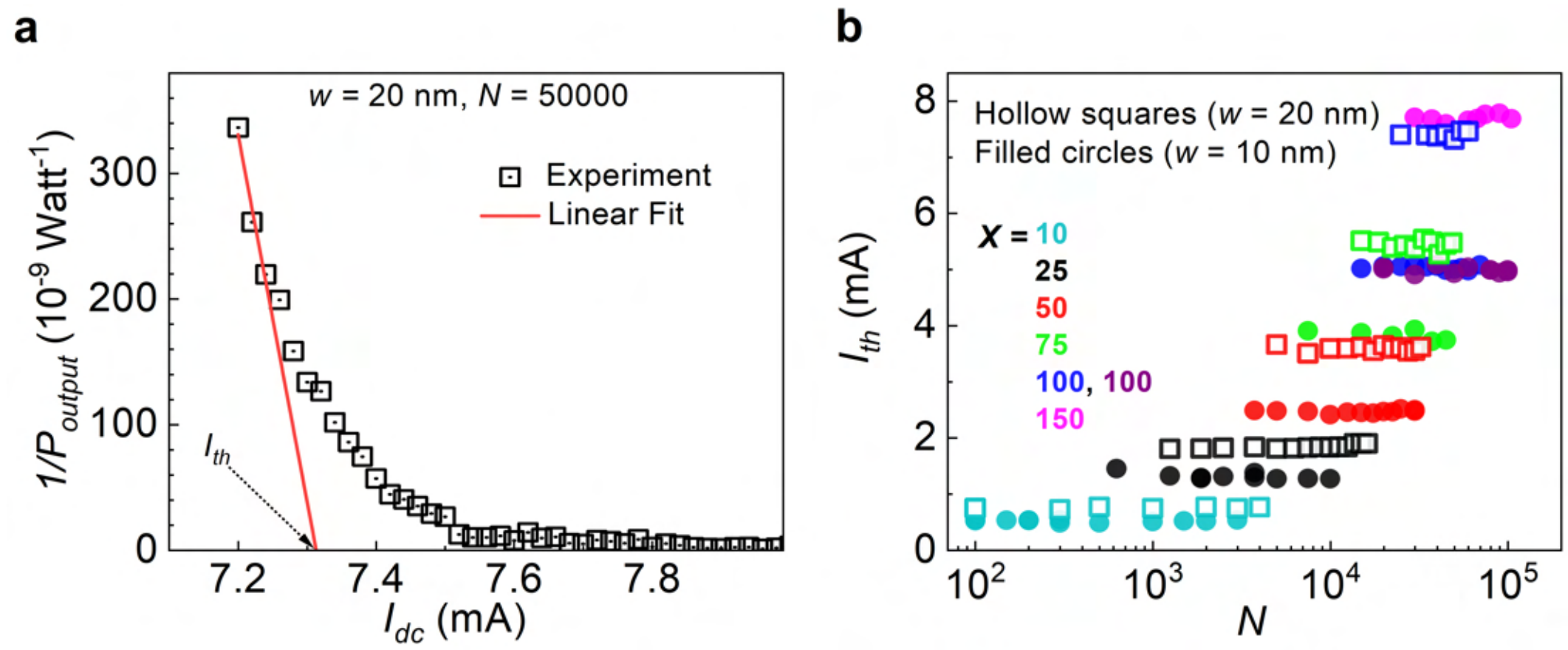

Figure S5: **Auto-oscillation threshold current. (a)** Illustration of auto-oscillation threshold current estimation via a linear fit of 1/*P* *vs.* I_{dc}. **(b)** Estimated *N* dependent auto-oscillation threshold current (I_{th}) for *w* = 10-nm (filled circles) and 20-nm (hollow squares) SHNO arrays.

The auto-oscillation threshold current density (J_{th}) is estimated using the equation:

$$J_{th} = I_{th}/(t \times w \times X) \tag{S4}$$

where t is the thickness and *w* the width of each nano-constriction. The resulting J_{th} values are shown in Fig. 2E in the main manuscript. The extracted threshold current density (J_{th}) remains constant across all array sizes and is only slightly different between the 10-nm and 20-nm arrays. The relative deviation from the average J_{th} is shown for each array in the top row of Fig. S6, with a relative standard deviation of 2.1% in the 20 nm arrays and 3.3% in the 10 nm arrays. The auto-oscillation frequency at threshold is similarly independent of array size, and even more tightly distributed, with a relative standard deviation of 1.0% in the 20 nm arrays and 1.1% in the 10 nm arrays (bottom row in Fig. S6).

2.4 Frequency tunability

The frequency tunability, also known as change of auto-oscillation frequency per unit current density, *df/dJ* is estimated from the change of frequency per unit current, i.e. *df/dI*$_{dc}$ $vs.$ I_{dc}, curve of the PSD plots. The inset of main Fig. 2f shows the exemplary plot of *df/dI*$_{dc}$ $vs.$ I_{dc} curve and its maximum average saturation point i.e. $(df/dI_{dc})_{max}$ as indicated in red solid line is used for estimating the *df/dJ*. Hence, *df/dJ* is estimated by using the

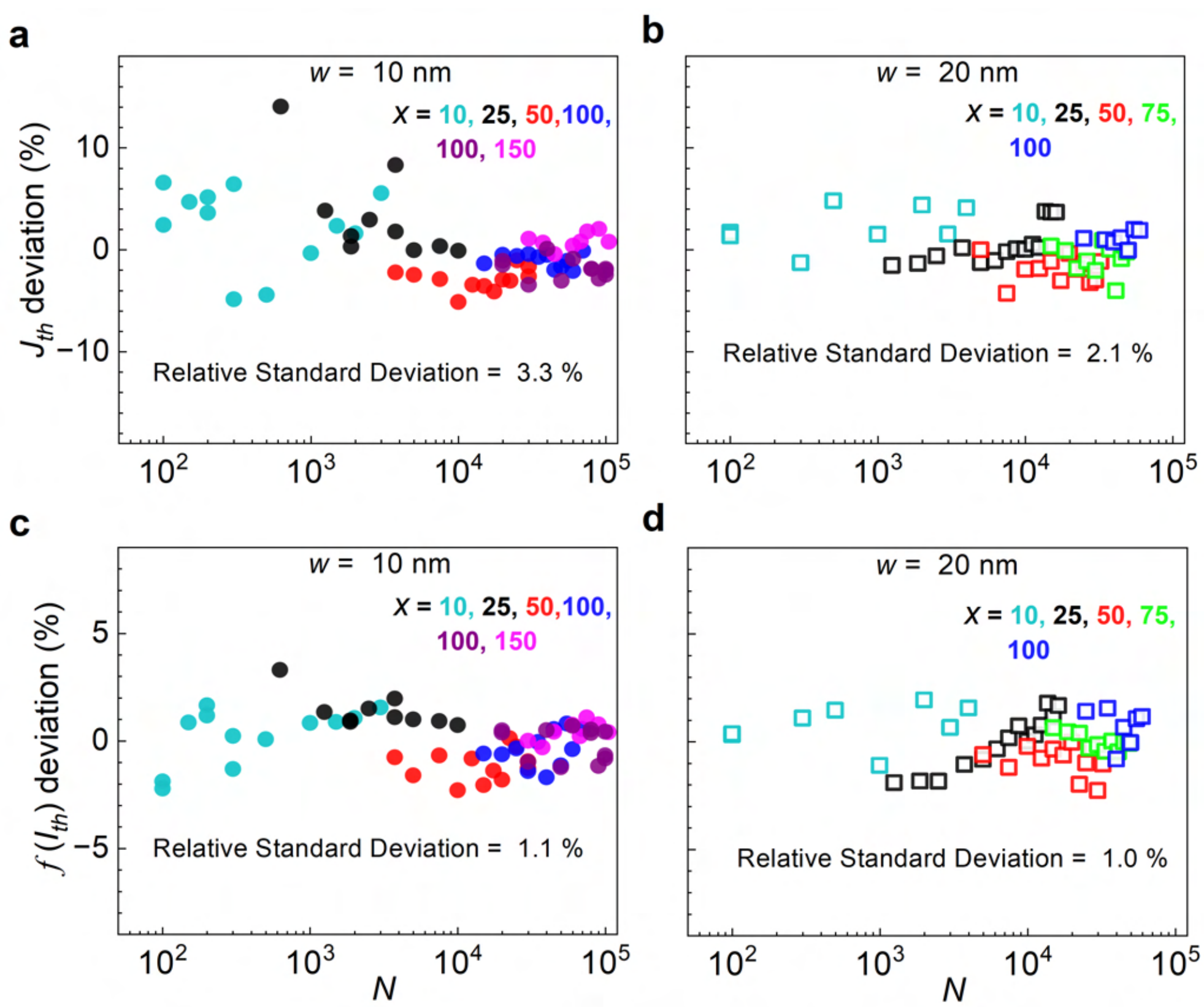

Figure S6: **Relative Standard Deviation of the threshold current and auto-oscillation frequency at threshold.** Estimated N dependent relative deviations from the average J_{th} & the average auto-oscillation frequency at threshold $f(I_{th})$ are shown for each array in the top and bottom rows, for w = 10-nm (filled circles) and 20-nm (hollow squares) SHNO arrays. The colors correspond to the same X values as in Fig. S5b.

equation:

$$df/dJ = (df/dI_{dc})_{max} \times t \times w \times X \tag{S5}$$

here df/dI_{dc} is the derivative of f vs. I obtained from the PSDs of each SHNO.

2.5 Record-high Quality factor

Fig. S7 shows a PSD plot for one of the largest 20-nm arrays ($N = 75 \times 650 = 48,750$ nano-oscillators) with the narrowest linewidth observed in all arrays. A new record-low linewidth of 25.3± 0.9 kHz at an auto-oscillation frequency of 26.2 GHz, corresponds to a new record-high quality factor for any spintronic oscillator of $Q = f/\Delta f = 1.04 \pm 0.04 \times 10^6$.

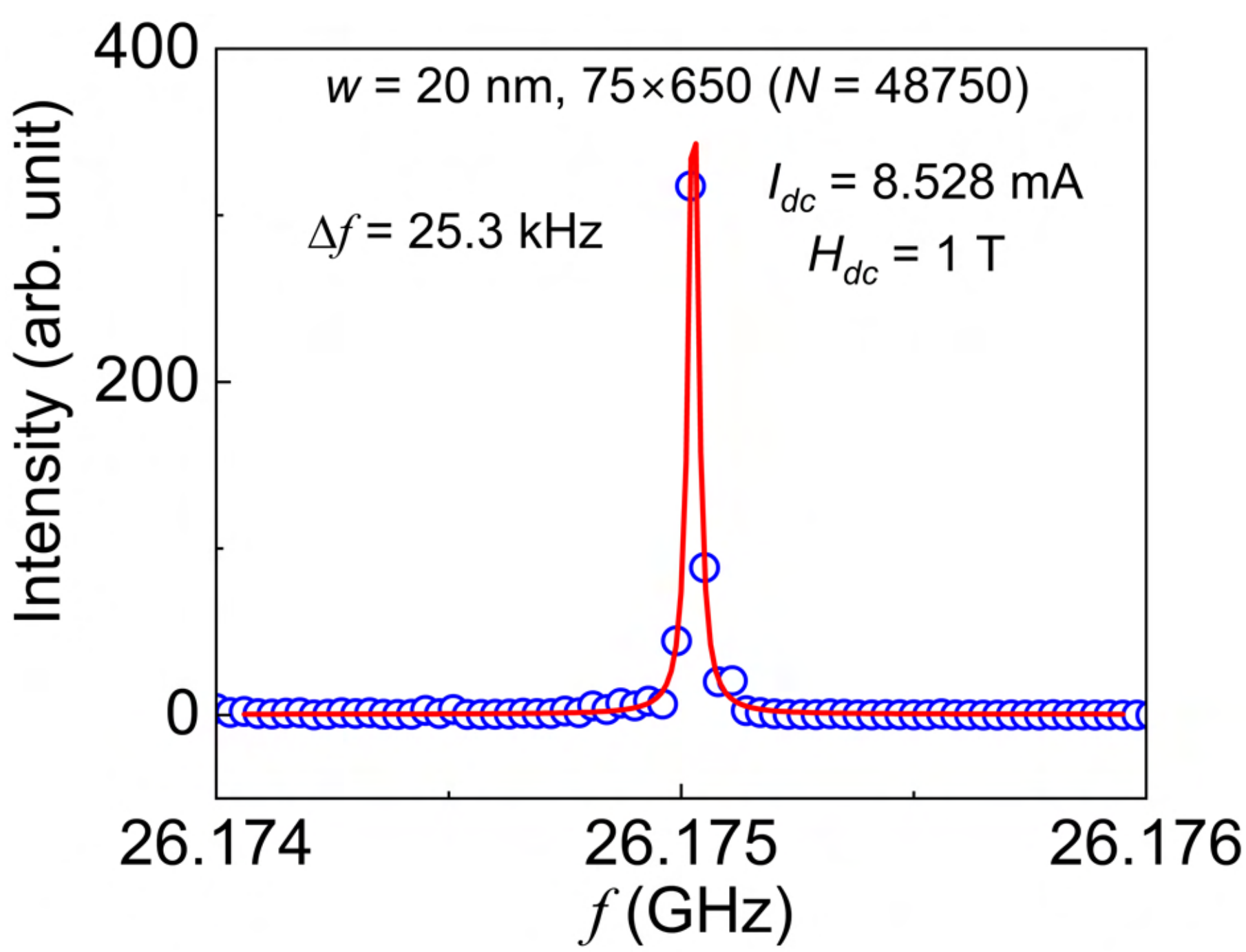

Figure S7: **Record high Quality factor.** The measured PSD (blue circles) of a 20-nm N = 48,750 array together with a Lorentzian fit (red solid line). The extracted linewidth 25.3±0.9 kHz results in a Q-factor of 1.04± 0.04 × 10^6.

2.6 Record-high SHNO auto-oscillation frequency

To test for higher frequency operation, we measured the PSD of a 20-nm N = 15000 (25 × 600) array in a field of 1.1 T. As can be seen in Fig. S8, a maximum frequency of 33.9 GHz was reached in this field, which is the highest recorded auto-oscillation frequency observed in SHNOs to date.

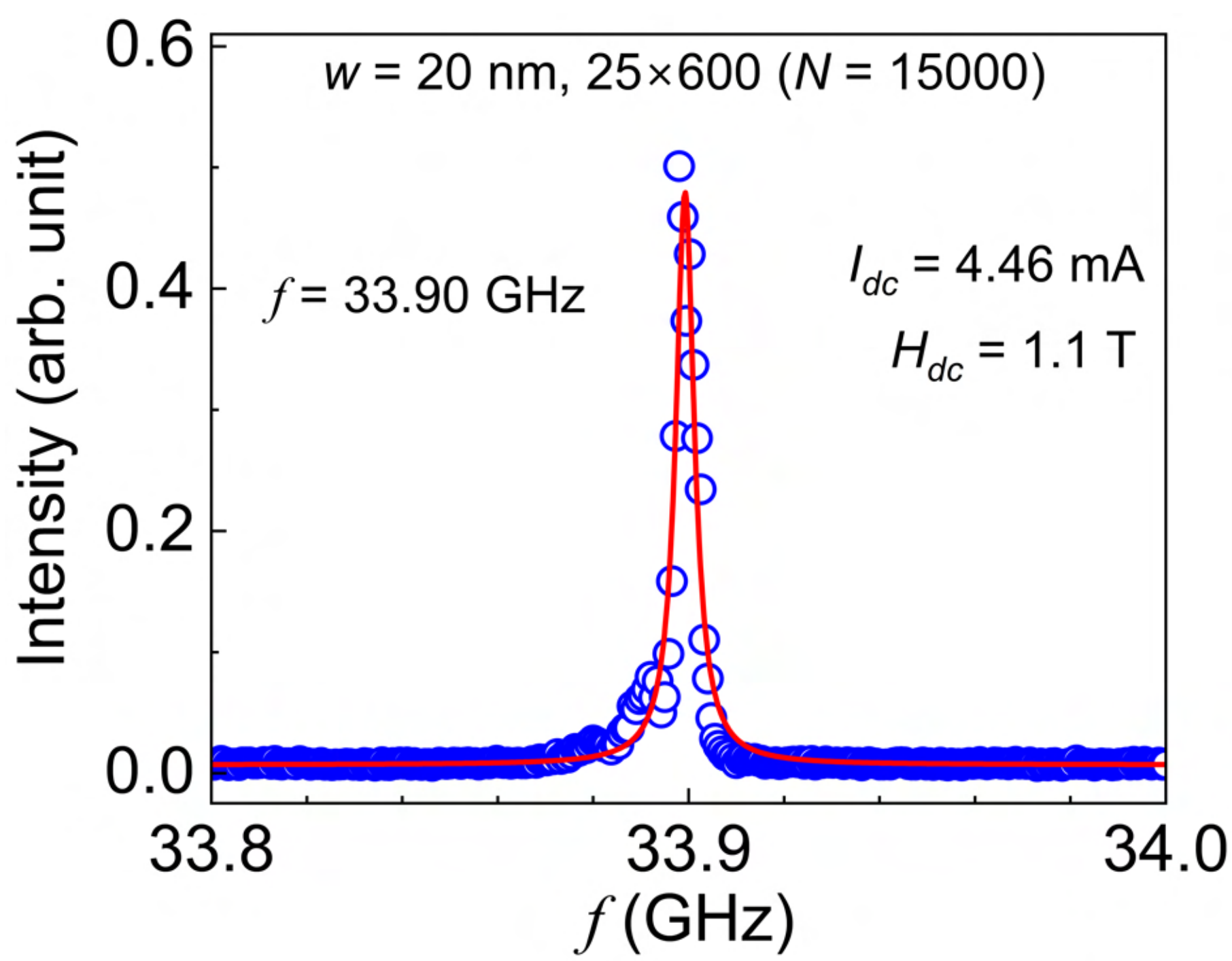

Figure S8: **Higher field (1.1 T) PSD.** The measured PSD (blue circles) at an applied field of 1.1 T, of a 20-nm N = 15000 (25 × 600) array together with a Lorentzian fit (red solid line). The extracted peak frequency is 33.90 GHz.

3 Simulations and modeling

3.1 Micromagnetic simulations

The micromagnetic simulations were performed by mumax^3 [2] for the array of 16×32 SHNOs for the geometry of the experimental samples with $w = 10$-nm, The grid contains 512×1536 cells of the size 0.75×0.75×1.4 nm each to accurately represent SHNOs cutouts. The array was supplemented with a mesa on both sides to avoid reflections of magnons back into the array area. This was achieved by quadratically increasing the Gilbert damping constant from the intrinsic value $\alpha = 0.024$ at the boundary of the array to $\alpha = 0.49$ at the edge of the mesa. The following magnetic parameters, extracted from the experimental results, were used: saturation magnetization $M_s = 936\text{kA/m}$, exchange stiffness $A_{ex} = 19\text{pJ/m}$, gyromagnetic ratio $\gamma/(2\pi) = 29.9$ GHz/T, perpendicular magnetic anisotropy $K_{u1} = 380\text{kJ/m}^3$.

3.2 The model of the magnon transfer

The measured profiles of the auto-oscillations can be explained by assuming each oscillator as an emitter of propagating spin waves, which exponentially decay over the distance with the decay length λ_{SW}. Our micromagnetic simulations reveal that SWs are propagating along the rows and columns of the array, and therefore, one can neglect the radial decay. Then along a column (in Y direction) the amplitude of a wave generated by $i-$th oscillator on $j-$th site can be written as:

$$a_{ij} = A\cos\left[2\pi f t - k_y \delta y\right]\exp(-2\pi\alpha_{eff} f y/v_y), \tag{S6}$$

where f is a frequency of SW, k_y and v_y are correspondingly components of a wavevector and group velocity along y, and $\delta y = (i-j)d$, with $d = 24$-nm – the distance between neighboring oscillators (center-to-center separation). Here we assumed $\lambda_{SW} = v_y/(2\pi\alpha_{eff} f)$.

Since the SHNOs array is synchronized, the total intensity on $j-$th site is:

$$I_j = \left[\sum_i A\exp(-2\pi\alpha_{eff} f|i-j|d/v_y)\cos(\delta\phi|i-j|)\right]^2, \tag{S7}$$

where $\delta\phi = k_y d$ is a phase shift between neighboring oscillators. For our case $\delta\phi \ll 1$, however the total phase

shift along the array can be substantial at high currents. Please note that the above equation is only valid for a synchronized array, since it adds SWs coherently [3]. Therefore, one should expect a different (broader) profile for unsynchronized arrays.

Substantially far from the edge of the array, i.e., when $j \gg \lambda_{sw}/d$, the summation in Eq. (S7) can be replaced by an infinite series. Neglecting the phase shift $\delta\phi$ between oscillators, one can get the maximum intensity $I_\infty = A^2 \coth^2 \frac{d}{2\lambda_{SW}}$. Therefore, for long enough arrays, the profile given by Eq. (S7) is flat over most of the array. However, near the edge, where the number of significant terms in a sum is limited, the intensity drops with the spatial slope defined by λ_{SW}. Thus, for relatively short arrays, the flat top disappears, and the profile has a pronounced maximum at the center.

The BLS measured profile can be expressed as the convolution of the ”intrinsic” profile Eq.(S7) and the Gaussian beam:

$$I_{BLS} = I_j * \exp\left(-\frac{(j-b)^2 d^2}{2\sigma^2}\right), \tag{S8}$$

where b is the position of the center of the BLS beam, and the dispersion coefficient $\sigma \simeq 200$ nm for our BLS setup.

One should notice that Eq.(S7) does not take into account the nonlinear behavior of the oscillations and, therefore, is valid only at low applied currents, near the threshold value. The fitting of BLS results by the above model is shown in Figure 3G of the main text. Surprisingly, this simple model works reasonably well even for high currents, where micromagnetic simulations show large amplitudes of the magnetization precession and a strongly nonlinear regime.

While both simulations and experiment show a linear dependence of the frequency on applied current for small arrays, it becomes quadratic for a large number of synchronized SHNOs, (see figure S3). Equation (S7) can provide insight into this dissimilarity, since the frequency of a fully synchronized array can be expressed as $f = f_0 + \mathcal{N}\bar{I}$, where f_0 is a frequency on the threshold and $\mathcal{N}$ is a coefficient of nonlinearity, which is positive for our case. It is useful to look at the dependence of I on λ_{SW}, since the spin wave decay length increases with the current alongside the velocity of magnons v_y. For simplicity, we can consider the intensity given by Eq. (S7) in the center

of the array with $2n+1$ rows:

$$I(\lambda_{SW}) = \left[\frac{1+\exp(d/\lambda_{SW}) - 2\exp(-dn/\lambda_{SW})}{-1+\exp(d/\lambda_{SW})}\right]^2. \tag{S9}$$

This dependence is close to linear in the case when the length of the array dn is comparable to a spin wave decay length λ_{SW}, i.e. for relatively small arrays, that is close to the dependence on the applied current given by the simulations and experimental measurements. However, for large n one can neglect the second term in the numerator, and the dependence becomes close to quadratic, which coincides with our experimental results. Thus, for large arrays the emission of spin waves predominantly increases the amplitude on other oscillators, while for a small number, it mainly contributes to the total damping.

Supplementary References

[1] V. Tiberkevich, A. Slavin, J.V. Kim, Microwave power generated by a spin-torque oscillator in the presence of noise. Appl. Phys. Lett. **91**(19), 192506 (2007)

[2] A. Vansteenkiste, J. Leliaert, M. Dvornik, M. Helsen, F. Garcia-Sanchez, B. Van Waeyenberge, The design and verification of MuMax3. AIP Adv. **4**(10), 107133 (2014)

[3] S. Demokritov, V. Demidov, O. Dzyapko, G. Melkov, A. Slavin, Quantum coherence due to bose–einstein condensation of parametrically driven magnons. New J. Phys. **10**(4), 045029 (2008)
